# Evaluating the influence of different anticoagulant blood collection tubes on the efficiency of circulating RNA extraction and Real-Time PCR quantification

**DOI:** 10.1007/s11033-026-12269-w

**Published:** 2026-08-21

**Authors:** Mauro Di Ruvo, Marco Benati, Gian Luca Salvagno, Laura Pighi, Veronica Li Vigni, Sara Visconti, Elisa Paviati, Flavia Sabbatini, Giuseppe Lippi

**Affiliations:** Section of Clinical Biochemistry and School of Medicine, University of Verona, P.le L.Scuro 37134, Verona, Italy

**Keywords:** Quality, Molecular biology, Preanalytical variability, miRNA, RNA

## Abstract

**Background and aims:**

Rigorous pre-analytical standardization is a prerequisite for reliable use of circulating microRNAs and RNA as clinical biomarkers. This study evaluates the influence of four common anticoagulants (sodium citrate, Dipotassium Ethylenediaminetetraacetic Acid (K2-EDTA), Lithium heparin, and Acid Citrate Dextrose (ACD)) on isolation and quantification of miRNA and RNA from human plasma.

**Materials and methods:**

Fifteen healthy volunteers were recruited from the research staff and donated blood to study the effects of different anticoagulants on molecular tests. After Trizol extraction, we assessed RNA quantification and purity via spectrophotometry and amplification efficiency via Real-Time PCR for miR-133a, cel-miR-39 (exogenous spike-in), and U6snRNA (endogenous control).

**Results:**

Our results confirmed that while K2-EDTA, sodium citrate and ACD provide comparable enzymatic environments for Real-time PCR, lithium heparin strongly inhibited reverse transcriptase and DNA polymerase activity, preventing successful amplification. Furthermore, specific differences in spectrophotometric purity ratios (A_260_/A_280_) were identified, suggesting that the chemical nature of the anticoagulant subtly influences the UV absorbance profile of the extract and its purity.

**Conclusions:**

For researchers focusing on circulating RNA and miRNA, sodium citrate, ACD and K2-EDTA should be regarded as suitable anticoagulants, with sodium citrate and ACD offering slightly better spectrophotometric purity profiles. Lithium heparin is strongly discouraged for studies involving enzymatic amplification (PCR), as the Trizol extraction method is insufficient to remove the inhibitory heparin molecules.

## Introduction

Reliable use of circulating biomarkers necessitates stringent pre-analytical standardization to minimize variability [1]. Liquid biopsy has emerged as a revolutionary tool in molecular diagnostics, with circulating miRNAs and RNA serving as highly stable indicators of physiological and pathological states [2]. However, the transition from clinical blood collection to molecular analysis is fraught with potential interference. Anticoagulants, necessary for plasma separation, can co-purify with nucleic acids and interact with downstream enzymatic processes [3]. In our previous article we found that underfilling of K_2_-EDTA (ethylenediaminetetraacetic acid) blood tubes may be a modest but analytically significant source of bias in molecular biology testing [4], however the acceptable recommendations, which are for different anticoagulant tubes in molecular assays associated with pre-analytic variables, are limited.

Zhelankin et al. [3] showed that the choice of anticoagulant in blood collection tubes is a pre-analytical factor that affects plasma c-miRNA profiles. They found that using EDTA blood collection tubes results in elevated hemolysis in plasma samples compared to other anticoagulants, leading to a shift in plasma c-miRNA profiles, primarily by changing the ratio between platelet-derived and RBC-derived miRNAs. They also found no significant differences in plasma c-miRNA profiles between the citrate-based tubes (Acid-Citrate-Dextrose (ACD), sodium citrate (NaCit) and Citrate, Theophylline, Adenosine, and Dipyridamole (CTAD)).

Leidinger et al. [5] showed that miRNA expression profiles can be affected by short-term storage in EDTA, with increasingly pronounced alterations observed during prolonged storage.

Therefore, aim of this study was to evaluate the impact of various common anticoagulants on miRNA/RNA profiling in order to identify which collection tubes enable the highest reliability. Among circulating microRNAs, miR-133a is one of the most extensively studied myomiRs due to its key role in cardiac and skeletal muscle physiology and its involvement in several pathological conditions, including myocardial infarction, heart failure, and muscular disorders. Notably, circulating levels of miR-133a are also influenced by physical activity, making it a particularly sensitive biomarker to pre-analytical variability. While several studies have evaluated the effects of different anticoagulants on circulating miRNA profiles, comparative analyses remain heterogeneous and often exclude ACD. Therefore, this study aimed to systematically compare multiple anticoagulants, including ACD, within the same experimental workflow [6].

## Materials and methods

### Sample collection and RNA extraction protocol

Fifteen healthy volunteers (6 men and 9 women with a mean age of 47.7 ± 12.2 years) were recruited from the research staff and donated blood for this work. All healthy volunteers were sampled at 8 a.m. None of the volunteers had engaged in physical activity during the previous 24 h, in accordance with previous evidence showing that circulating miR-133a levels are transiently influenced by exercise and tend to return toward baseline within 24 h after physical activity [7]. The study was approved by the Ethics Committee of the University Hospital of Verona (970CESC; July 20, 2016) and was conducted in accordance with the relevant guidelines and regulations. Written informed consent was obtained from all participants. Blood was collected from each donor in tubes containing the anticoagulants: Lithium Heparin (Li-Hep), Sodium Citrate (NaCit), ACD and K2-EDTA (BD Vacutainer® tubes, Becton Dickinson and Company, Franklin Lakes, NJ, USA). Plasma was isolated via centrifugation at 3500 g for 10 min at 4 °C and immediately processed or stored at -80 °C to prevent RNA degradation. Centrifugation of plasma for microRNA (miRNA) studies is recommended to be performed within 2 h of blood collection to minimize cell contamination and hemolysis. While 4 °C is traditionally preferred to minimize RNase activity, studies have shown that centrifugation at room temperature (RT) or 4 °C yields comparable results for most plasma miRNA expression profiles, provided that the blood is processed within 2 h of collection [8].

Total RNA, including the small RNA fraction, was extracted from 250 µL of plasma using the Trizol™ LS (Thermo Fisher Scientific) reagent, according to the manufacturer’s specifications [9]. During the lysis phase, an exogenous spike-in (cel-miR-39) was added to monitor extraction efficiency. RNA concentration and purity (A_260_/A_280_ and A_260_/A_230_ ratios) were assessed using a NanoDrop 2000 Spectrophotometer (Thermo Scientific, Wilmington, DE, USA).

### Real-time PCR (qPCR)

The RNA obtained from each anticoagulant tube, was analyzed using a quantitative real time RT-PCR to detect (a) miR-133a, a muscle-specific miRNA; (b) U6snRNA, a small nuclear RNA used as an internal reference; and (c) cel-miR-39, used as a recovery control (mirBase sequence information number MI0000010).

TaqMan real-time quantitative PCR amplification reactions were carried out in an AB 7500 fast Real-Time system following “Taqman™ Small RNA assay” and the recommended thermal conditions (Applied Biosystems, Thermo Fisher Scientific, Massachusetts, USA). For each individual donor, all blood collection tubes were processed in parallel under identical conditions to minimize pre-analytical variability.

### Statistical analysis

Differences in RNA concentration were reported as mean, while A_260_/A_280_ ratio, A_260_/A_230_ ratio, and Cycle threshold (Ct) between the various anticoagulant tubes were reported as median ± interquartile range (IQR). To assess statistical significance, the Wilcoxon signed-rank test was employed, as it is specifically designed for non-parametric paired data, while graphs were obtained using Friedman test. Statistical significance was defined as *p* < 0.05. All descriptive statistics and analyses were performed using GraphPad Prism software (La Jolla, CA, USA, version 6.01, 21 September 2012).

## Results

### Spectrophotometric purity profiles

**Fig. 1:**
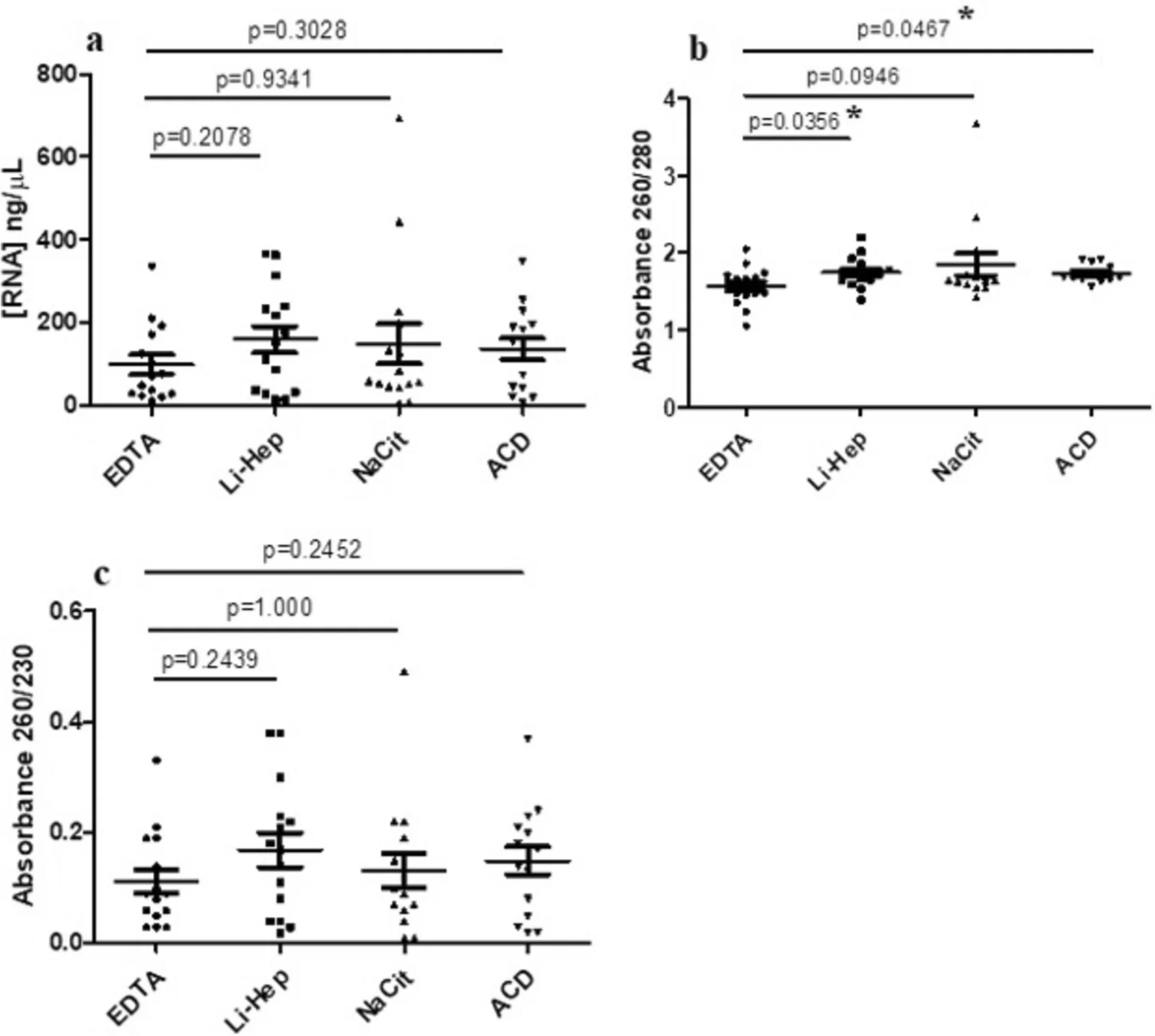
Influence of anticoagulant type tube on the RNA. (**a**) RNA concentration (ng/µL) across different anticoagulant blood collection tubes; (**b**) A260/A280 purity ratios; (**c**) A260/A230 purity ratios (* p < 0.05)

RNA quantification revealed high yields across all groups with generally high purity (Table 1). However, a significant discrepancy was observed in the A_260_/A_280_ ratios (Fig. 1), which can indicate protein or chemical contamination. Specifically, EDTA samples showed an average ratio of ~ 1.5, whereas NaCit, LiHep, and ACD samples exhibited higher ratios that more closely approached the ideal value of 2.0. This lower ratio in the K2-EDTA group was statistically significant when compared to LiHep (*p* = 0.0356) and ACD (*p* = 0.0467). Conversely, no significant differences were observed in the A_260_/A_230_ ratios across the different groups.

**Table 1:** Mean RNA concentration (ng/µL) and median spectrophotometric purity ratios ± IQR (Interquartile Range) across different anticoagulant blood collection tubes from fifteen healthy donors (* p < 0.05)

	EDTA	Li-Hep	NaCit	ACD
RNA (ng/µL)	93.08	126.15	188.23	100.71
A_260_/A_280_ ratio ± IQR	1.56* ± 0.24	1.71 ± 0.17	1.64 ± 0.18	1.71 ± 0.12
A_260_/A_230_ ratio ± IQR	0.09 ± 0.11	0.17 ± 0.165	0.1 ± 0.105	0.17 ± 0.14

### PCR interference and Cycle thresholds (Ct)

**Fig. 2:**
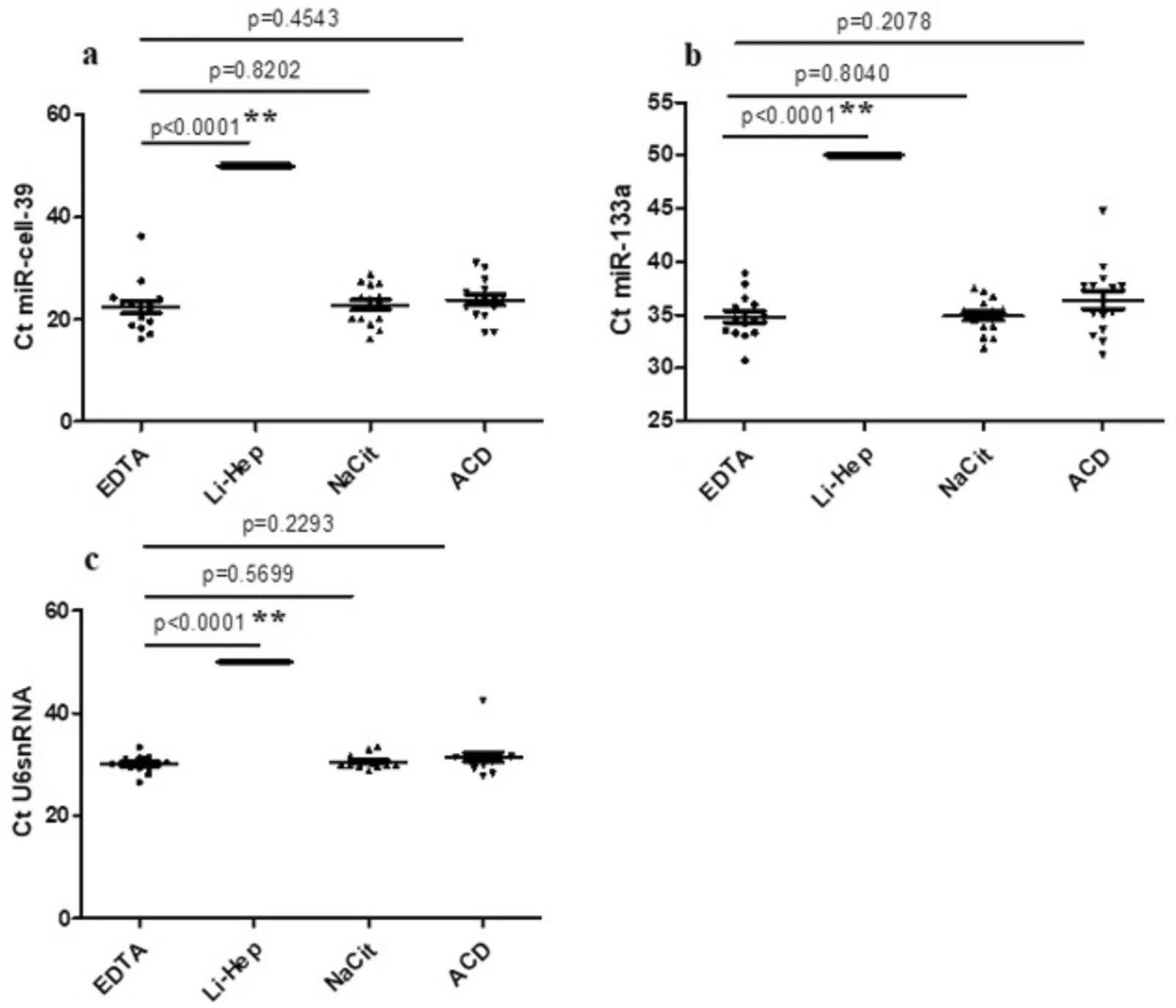
Variability in Real-time PCR Cycle Thresholds (Ct) by Anticoagulant blood collection tube (** p < 0.0001) for (**a**) cel-miR-39, (**b**) miR-133a and (**c**) U6snRNA

The most striking results were observed during the amplification phase. K2-EDTA, NaCit, and ACD all facilitated successful amplification for all targets. As shown in Table 2, the Ct values were consistent across these three media with no statistically significant differences, suggesting they are equally effective for RNA/miRNA downstream applications. Conversely, all heparinized sample resulted in Ct values for miR-133a, cel-miR-39, and U6snRNA > 50, rendering them effectively undetermined (Fig. 2). These results confirm a total failure of the polymerase enzyme to replicate the target sequences in the presence of LiHep.

**Table 2:** Quantitative RT-PCR analysis of three miRNA/RNA targets

Target	EDTA	Li-Hep	NaCit	ACD
cel-miR-39 (Ct ± IQR)	22.48 ± 4.52	N/A	23.51 ± 5.47	23.9 ± 3.90
miR-133a (Ct ± IQR)	34.68 ± 2.40	N/A	35.12 ± 1.85	36.99 ± 3.5
U6snRNA (Ct ± IQR)	30.06 ± 1.40	N/A	30.16 ± 0.93	31.09 ± 1.52

Data represent median Cycle Threshold (Ct) values ± IQR (Interquartile Range) across various anticoagulant blood collection tubes from fifteen healthy donors.

## Discussion

Blood is an abundant and readily accessible source of biological material used for diagnosing and monitoring diseases. It is routinely drawn from both healthy individuals and patients due to the minimally invasive nature of the procedure. EDTA coated tubes are the conventional containers for blood collection in molecular testing [10]. EDTA and citrate salts are considered the most effective anticoagulants to extract of high molecular weight nucleic acid. Since they have.

very similar effects on PCR analysis [11] and collection tubes with EDTA are used more frequently, in our recent study we recommended K2-EDTA as the anticoagulant of choice. Moreover, samples do not need to be rejected if the blood tube is underfilled by 75% or lower [4].

The failure of LiHep in PCR applications is well-documented in the literature [12], but remains a critical issue in clinical research. Heparin is a highly charged polysaccharide that mimics the structure of the DNA backbone. Due to this structural similarity, it is often co-purified during the Trizol extraction process. Once in the reaction mix, it binds to the Taq polymerase, competitively inhibiting its activity. Heparinase treatment should be added to the extraction protocols [13]. However, the degree of heparin inhibition depends on its concentration in the nucleic acid preparation [14], and can be influenced by magnesium ion concentration, DNA dilution, polymerase type and enzyme amount [15]. This is consistent with the results of our study, which found that samples collected with Li-Hep tube had no amplification compared to those collected with other anticoagulants.

Regarding the purity data, the significantly lower A_260_/A_280_ ratio in K2-EDTA (~ 1.5) compared to NaCit, ACD and LiHep (~ 2.0) is noteworthy. A ratio of ~ 1.5 is often considered "not acceptable" for RNA [16], and may indicate higher retention of certain plasma proteins or slightly shifts absorbance spectrum due to the salt. However, since the quantitative RT-PCR results for K2-EDTA were identical to those of NaCit and ACD, this lower purity ratio does not appear to translate into functional inhibition of the PCR reaction.

The results of this study underscore the critical importance of pre-analytical standardization in the burgeoning field of liquid biopsy and circulating biomarker analysis. Our findings demonstrate that the choice of anticoagulant is not merely a procedural detail, but a decisive factor that can determine the viability of molecular diagnostics. While NaCit, ACD, and K_2_-EDTA all proved to be reliable media for the downstream quantification of circulating miRNAs and RNA, they exhibited distinct biochemical profiles. Specifically, NaCit and ACD provided superior spectrophotometric purity, yielding A_260_/A_280_ ratios closer to the ideal 2.0 value. Although K_2_EDTA produced slightly lower purity ratios (averaging 1.5), this did not translate into functional inhibition during the amplification phase, as evidenced by consistent Ct values across these three groups. This suggests that while K2-EDTA may reflect differences in residual contaminants or absorbance profiles, it remains a reliable anticoagulant for downstream PCR-based applications. Conversely, our data reinforce a strict contraindication for LiHep in studies involving enzymatic amplification. The complete failure of PCR amplification in heparinized samples (resulting in effectively undetermined Ct values) confirms that standard Trizol-based extraction methods are insufficient to eliminate heparin’s potent inhibitory effects. Given that heparin competitively binds to Taq Polymerase by mimicking the DNA backbone, its presence renders samples useless for molecular profiling unless costly and time-consuming enzymatic treatments (e.g., heparinase) are employed.

Regarding miR-133a, a well-known and clinically relevant biomarker for various cardiac diseases including NSTEMI, our study suggests that sodium citrate is the preferred anticoagulant when highly precise and accurate quantification is required, as it exhibited the lowest preanalytical variability [17].

We acknowledge that this study has some limitations, including the small sample size and the lack of electrophoretic evaluation of nucleic acid integrity (RIN). Another limitation of the study is the inability to determine the degree of hemolysis in the different blood collection tubes. Further studies involving a larger number of samples will be required to evaluate RIN values and hemolysis levels, as well as to extend our findings to other real-time PCR assays used in clinical medicine.

## Conclusions

For Researchers and clinicians aiming to integrate miRNA/RNA profiling into routine practice, NaCit and ACD offer the highest purity, while K2-EDTA remains a highly functional and versatile alternative. Future research should focus on larger cohorts and include electrophoretic assessments, such as the RIN, to further refine these pre-analytical guidelines and ensure that the "liquid biopsy" can transition from a promising research tool to a reliable clinical application.

## Data Availability

No datasets were generated or analysed during the current study.

## Abbreviations

**K**_**2**_**-EDTA**: K_2_-Ethylenediaminetetraacetic acid
**ACD**: Acid Citrate Dextrose
**Li-Hep**: Lithium heparin
**NaCit**: Sodium citrate
**NaF-K**_**2**_** Ox**: Sodium fluoride as stabilizer and the additive Potassium oxalate as anticoagulant
**RNA**: Ribonucleic Acid
**DNA**: Deoxyribonucleic Acid
**A**_**260**_**/A**_**230**_** ratio**: 260/230 Absorbance ratio
**A**_**260**_**/A**_**280**_** ratio**: 260/280 Absorbance ratio

## References

[1] Plebani M (2018) Harmonization in laboratory medicine: more than clinical chemistry? Clin Chem Lab Med. 10.1515/cclm-2017-0865 29095697 10.1515/cclm-2017-0865

[2] Metcalf GAD (2024) MicroRNAs: circulating biomarkers for the early detection of imperceptible cancers via biosensor and machine-learning advances. Oncogene. 10.1038/s41388-024-03076-3 38839942 10.1038/s41388-024-03076-3 PMC11226400

[3] Zhelankin AV, Iulmetova LN, Sharova EI (2022) The impact of the anticoagulant type in blood collection tubes on circulating extracellular plasma microRNA profiles revealed by small RNA sequencing. Int J Mol Sci. 10.3390/ijms231810340 36142259 10.3390/ijms231810340 PMC9499385

[4] Benati M, Pighi L, Paviati E, Visconti S, Lippi G, Salvagno GL (2024) Preanalytical impact of incomplete K2EDTA blood tube filling in molecular biology testing. Diagnostics (Basel). 10.3390/diagnostics14171934 39272719 10.3390/diagnostics14171934 PMC11394328

[5] Leidinger P, Backes C, Rheinheimer S, Keller A, Meese E (2015) Towards clinical applications of blood-borne miRNA signatures: the influence of the anticoagulant EDTA on miRNA abundance. PLoS ONE. 10.1371/journal.pone.0143321 26599228 10.1371/journal.pone.0143321 PMC4658123

[6] Mussbacher M, Krammer TL, Heber S, Schrottmaier WC, Zeibig S, Holthoff HP, Pereyra D, Starlinger P, Hackl M, Assinger A (2020) Impact of anticoagulation and sample processing on the quantification of human blood-derived microRNA signatures. Cells 9(8):1915. 10.3390/cells9081915 32824700 10.3390/cells9081915 PMC7464075

[7] Cui S, Sun B, Yin X, Guo X, Chao D, Zhang C, Zhang CY, Chen X, Ma J (2017) Time-course responses of circulating microRNAs to three resistance training protocols in healthy young men. Sci Rep 7(1):2203. 10.1038/s41598-017-02294-y 28526870 10.1038/s41598-017-02294-y PMC5438360

[8] Zendjabil M (2024) Preanalytical, analytical and postanalytical considerations in circulating microRNAs measurement. Biochem Med (Zagreb). 10.11613/BM.2024.020501 38882585 10.11613/BM.2024.020501 PMC11177657

[9] TRIzol LS Reagent User Guide (Pub. No. MAN0000806). Thermo Fisher Scientific. https://documents.thermofisher.com/TFS-Assets/LSG/manuals/trizol_ls_reagent.pdf.

[10] Nguyen LT, Pollock CA, Saad S (2024) Extraction of high quality and high yield RNA from frozen EDTA blood. Sci Rep. 10.1038/s41598-024-58576-9 38622175 10.1038/s41598-024-58576-9 PMC11018810

[11] Yamagata H, Kobayashi A, Tsunedomi R, Seki T, Kobayashi M, Hagiwara K, Chen C, Uchida S, Okada G, Fuchikami M, Kamishikiryo T, Iga JI, Numata S, Kinoshita M, Kato TA, Hashimoto R, Nagano H, Okamoto Y, Ueno S, Ohmori T, Nakagawa S (2021) Optimized protocol for the extraction of RNA and DNA from frozen whole blood sample stored in a single EDTA tube. Sci Rep. 10.1038/s41598-021-96567-2 34426633 10.1038/s41598-021-96567-2 PMC8382694

[12] Holland NT, Smith MT, Eskenazi B, Bastaki M (2003) Biological sample collection and processing for molecular epidemiological studies. Mutat Res. 10.1016/s1383-5742(02)00090-x 12787814 10.1016/s1383-5742(02)00090-x

[13] Izraeli S, Pfleiderer C, Lion T (1991) Detection of gene expression by PCR amplification of RNA derived from frozen heparinized whole blood. Nucleic Acids Res. 10.1093/nar/19.21.6051 1719488 10.1093/nar/19.21.6051 PMC329069

[14] Ding M, Bullotta A, Caruso L, Gupta P, Rinaldo CR, Chen Y (2011) An optimized sensitive method for quantitation of DNA/RNA viruses in heparinized and cryopreserved plasma. J Virol Methods. 10.1016/j.jviromet.2011.05.012 21645549 10.1016/j.jviromet.2011.05.012 PMC3143304

[15] Cai D, Behrmann O, Hufert F, Dame G, Urban G (2018) Direct DNA and RNA detection from large volumes of whole human blood. Sci Rep. 10.1038/s41598-018-21224-0 29467420 10.1038/s41598-018-21224-0 PMC5821888

[16] Assessment of Nucleic Acid Purity, Technical Note 52646, Thermo Fischer Scientific. https://documents.thermofisher.com/TFS-Assets/CAD/Product-Bulletins/TN52646-E-0215M-NucleicAcid.pdf

[17] Wexler Y, Nussinovitch U (2020) The diagnostic value of Mir-133a in ST elevation and non-ST elevation myocardial infarction: a meta-analysis. Cells 9(4):793. 10.3390/cells9040793 32218383 10.3390/cells9040793 PMC7226415

